# Laparoscopic Management of Chylous Leakage Using a Direct Lymph Node Injection with Methylene Blue as a Leakage Point Location Strategy in a Patient with Retroperitoneal Extragonadal Seminoma

**DOI:** 10.1089/cren.2018.0056

**Published:** 2018-09-20

**Authors:** José Daniel Subiela, Josep Balañà, Amad Abu-SubohAbadia, Julio Francisco Calderón, Asier Mercadé, José Salvador Bayarri, Antonio Rosales Bordes

**Affiliations:** ^1^ Department of Urology, Fundació Puigvert, Universitat Autònoma de Barcelona, Barcelona, Spain.; ^2^ Department of Radiology, Fundació Puigvert, Universitat Autònoma de Barcelona, Barcelona, Spain.

**Keywords:** chyle leakage, laparoscopic management, intranodal lymphangiography, extragonadal seminoma

## Abstract

***Background:*** The first-line treatment in cases of chylous leakage is conservative, and operation should be considered only in patients who fail to respond to this treatment. The main clinical concern is the difficulty of intraoperative localization of the site of leakage that can affect surgical outcome.

***Case Presentation:*** A 33-year-old man presented with a 4-month history of abdominal pain and weight loss. CT scan revealed enlarged retroperitoneal lymph nodes. Retroperitoneal lymph node biopsy was performed owing to the suspicion of lymphoproliferative disease, with a pathological result of nonspecific adenitis. Because of persistence of pain, an abdominal CT scan showed a large left retroperitoneal fluid collection that was found to be compatible with chyle after drainage. Conservative treatment was established, but because of its failure, surgical management was attempted by the laparoscopic approach. Intraoperative direct lymph node injection of methylene blue was used as a leakage point location strategy that allows selective ligation of the site of leakage. Thereafter a gradual reduction in chyle output to zero was observed.

***Conclusion:*** The laparoscopic approach could be a feasible and successful method for the management of chyle leakage in patients refractory to conservative treatment. Intraoperative direct lymph node injection of methylene blue could be a useful technique to facilitate detection of the site of leakage during operation.

## Introduction

Chyle leakage is a rare but severe complication that can occur after different surgical procedures.^1^ Without treatment, chyle leakage can generate hypoproteinemia, edema, malnutrition, and decreased immunologic function, increasing the risk of sepsis and even death.^2^ Conservative treatment with dietary adjustments (low fat, medium-chain triglycerides, high protein, and low salt), octreotide, and diuretics represents the first-line approach.^3^ Nowadays, lymphangiography with embolization is recognized to be a useful method with acceptable outcomes.^1^ However, patients with high drainage output who do not respond to conservative treatment are candidates for surgical treatment.^1,2^ The main clinical concern is the difficulty of intraoperative localization of the site of leakage that can compromise the success of operation. In this study we present a case of refractory retroperitoneal chyle leakage in a patient with an extragonadal retroperitoneal seminoma that was successfully managed laparoscopically using intraoperative lymphangiography with direct lymph node injection of methylene blue as a leakage site location strategy.

## Case Report

A 33-year-old man presented with a 4-month history of abdominal pain and weight loss. On examinations, all parameters were within normal limits. Therefore, an abdominal CT scan showed the presence of retroperitoneal enlarged lymph nodes without other pathological findings. Retroperitoneal lymph node biopsy by the laparoscopic approach was then performed because of the suspicion of lymphoproliferative disease, and the pathological result was nonspecific adenitis.

**FIG. 1.**
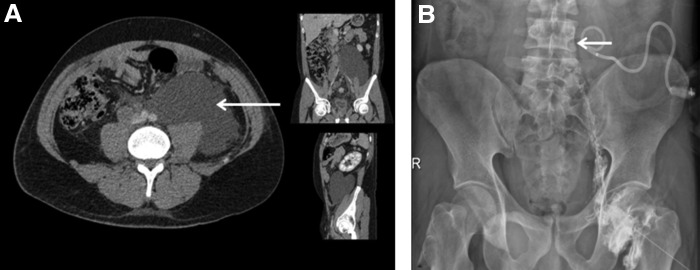
**(A)** Abdominal CT scan axial, coronal, and sagittal showing retroperitoneal chyle leak (*arrow*). **(B)** Lymphangiography shows the extravasated iodixanol (*arrow*) at fourth lumbar spine level within retroperitoneum.

Because of persistent abdominal pain, in association with left inguinal pain, a further abdominal CT scan was performed. This showed a large left retroperitoneal fluid collection (Fig. 1A) that caused slight left hydronephrosis and retroperitoneal lymph node enlargement in the interaortocaval, precaval, and right common iliac artery territories. In the presence of these findings, percutaneous drainage of the collection was carried out. With the patient in the supine position, under local anesthesia (10 mL of 2% mepivacaine), the lesion was punctured with a 21-gauge Chiba needle under ultrasonography and fluoroscopic guidance, leaving an 8.5F pigtail drainage catheter (Ultrathane^®^ drainage catheter set; Cook, Inc.) without complications.

The drainage recovered 1100 mL of milky liquid. Analysis revealed high levels of protein and triglycerides compatible with chyle, raising suspicion of cisterna chyli injury because of a history of retroperitoneal operation. Conservative management with a low-fat diet, medium-chain triglycerides, and octreotide was established, but the pigtail output was maintained at 1500 to 2000 mL per day for 2 weeks. For these reasons, the decision was taken to use surgical management.

**FIG. 2.**
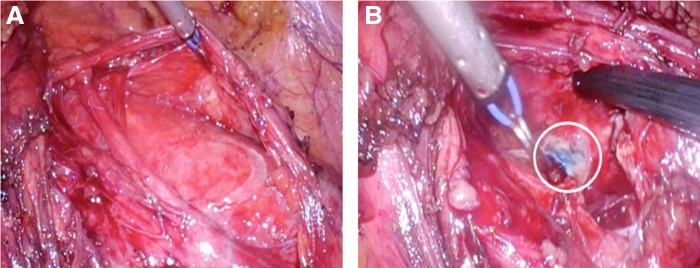
**(A)** Draining sinus in retroperitoneal location before direct Injection of inguinal lymph node with methylene blue. **(B)** Draining sinus 4 minutes after direct injection of inguinal lymph node with methylene blue (*white circle* shows the leak point as a *blue spot*).

During surgical planning, lymphangiography was performed after gaining direct access to the inguinal lymph node under ultrasonography guidance using a 22-gauge spinal needle. Iodixanol 270 mg/mL contrast (Visipaque™; GE Healthcare) was injected and the radiographic image showed perfect opacification of the lymphatic afferent and efferent vessels of the punctured lymph node and contrast leakage at the level of the fourth lumbar spine, within the retroperitoneum (inferior to the cisterna chyli) (Fig. 1B). A laparoscopic transperitoneal approach to the retroperitoneum was used. Trocar disposition was as follows: paraumbilical (10 mm), hypochondrium (5 mm), iliac fossa (12 mm), and anterior axillary line (5 mm). Dissection of the white line of Toldt was carried out until access was gained to the retroperitoneum. After identification of the chyle collection (Fig. 2A), the interventional radiologist performed intraoperative lymphography through direct inguinal lymph node puncture (as described previously) to corroborate the findings and then proceeded to the injection of 5 mL methylene blue in the cannulated lymph node. Four minutes later, leakage of methylene blue in the retroperitoneum (Fig. 2B) was identified, with localization of the leakage point. Selective ligation was performed using a 3-0 polyglactin suture, encompassing the area of leakage, and *n*-butyl cyanoacrylate was applied after stitching. Pathological lymph nodes were identified and extirpated for pathological analysis.

After operation, the drainage output diminished from ∼1500 to 2000 mL per day to <500 mL per day. Pathological evaluation of the adenopathies resulted in a diagnosis of seminoma. Testicular ultrasonography was carried out without pathological findings and the diagnosis of extragonadal retroperitoneal seminoma was considered. Chemotherapy using a cisplatin plus etoposide regimen was instituted; in addition, the patient remained under conservative treatment for chyle leakage, with gradual reduction of pigtail output to zero at 2 weeks after operation.

## Discussion

Chyle leakage is a rare condition that in most cases occurs secondary to unsuspected injury of the cisterna chyli or its branches during abdominal or retroperitoneal operation.^2,3^ Lymphadenectomy is the most frequent procedure associated with this complication.^1^

The most common symptom is abdominal distension, but pain, peritonitis, and chylous ascites may occur days after operation.^1–3^ The analysis of fluid is the most important diagnostic instrument, but the fluid characteristics used to diagnose chyle leakage differ between reports. A recent literature review defined chyle as a milky, cloudy, and turbid liquid with a triglyceride level >200 mg/dL^3^; however, the identification of chylomicrons represents the gold standard for diagnosis.^1^

CT scan and ultrasonography are useful imaging modalities for identification of intra-abdominal chyle accumulations, but both fail to identify the site of leakage.^2^ Lymphangiography and lymphoscintigraphy are the most widely used and accurate techniques to confirm the diagnosis and localize the site of leakage.^1,2^

As mentioned above, once the correct diagnosis has been established, conservative treatment with dietary adjustments, octreotide, and diuretics represents the first-line approach, with a success rate of 80%.^1^ Many management approaches have been described in patients with chyle leakage refractory to such conservative treatment. Recently, it has been reported that bipedal lymphangiography with Lipidol represents a promising method to close the leakage, with a success rate of ∼70% in patients with a drainage volume of <500 mL per day.^1,2^ Another minimally invasive method is percutaneous embolization using *n*-butyl cyanoacrylate that could be useful as an early radiology intervention in severe cases of postoperative chyle leakage.^1,2^ The implantation of transjugular intrahepatic portosystemic or peritoneovenous shunts has also been described; however, this approach is limited to patients with severe chyle leakage who are poor candidates for operation.^2^ Operation is reserved for patients with chylous leak >1000 mL per day for >5 days, patients who fail to respond to conservative treatment for >2 weeks, and patients who have short-term relapses.^1,2^

When operation is indicated, open procedure remains the gold standard for management of chyle leakage; however, the laparoscopic approach is increasingly used because it has been reported to offer magnification of the surgical field and to improve specific identification of the site of leakage.^4^ Although intraoperative localization of the site of leakage is the principal concern during the planning of operation because it is critical to a positive outcome, information on technical details is lacking.

Previous studies have described the oral administration of foods rich in lipids before operation (2–6 hours previously), such as butter cream, peanut oil, liquid whipping cream colored with patent blue dye, and milk^4^; this results in a change in the color of the chyle, improving identification of the site of leakage. However, despite such preparation, careful exploration of the suspected site is needed that can lead to confusion in cases of multiple or large leakages. In the patient reported here, use of intraoperative lymphangiography with direct lymph node injection of methylene blue permitted identification of the specific site of the leak as a blue point in the surgical field, thereby allowing selective ligation of the involved lymphatic branch.

A further consideration to be borne in mind when using the laparoscopic approach for management of chyle leakage is the possibility of an additional leakage point; in this regard the use of fibrin glue has been reported to achieve complete stasis of the lymphatic system.^4^ In the present case, use of *n*-butyl cyanoacrylate proved effective in avoiding additional chyle leakage.

In conclusion, the laparoscopic approach could be a feasible and successful method for the management of chyle leakage after operation in patients refractory to conservative treatment. We propose intraoperative lymphangiography with direct lymph node injection of methylene blue as a reasonable alternative to achieve specific localization of the site of leakage during operation, allowing selective ligation of the involved lymphatic branch.

## Abbreviation Used

CT: computed tomography
